# Editorial: Keratoconus – What We Do Not Know

**DOI:** 10.2174/1874364101711010173

**Published:** 2017-07-31

**Authors:** Zisis Gatzioufas, Samer Hamada, Damian Lake, Berthold Seitz

**Affiliations:** 1 Queen Victoria Hospital, Corneo-Plastic Unit East Grinstead, United Kingdom; 2 Dpt. of Ophthalmology, Saarland University Hospital, Homburg/Saar, Germany

Keratoconus has been recognized and investigated for more than 150 years [1]. Especially over the last decades, there has been intensive translational and clinical research in the field of corneal ectatic diseases, thereby revolutionizing the diagnosis and management of keratoconus. However, despite fundamental advances in understanding the complexity of this entity, the true nature of keratoconus remains merely unknown.

Historically, ophthalmologists have described keratoconus as a progressive, non-inflammatory disorder of the cornea, associated with corneal steepening and thinning [1, 2]. The Global Panel on Keratoconus and Ectatic Diseases recently stated that ‘abnormal posterior ectasia, abnormal corneal thickness distribution and clinical non-inflammatory corneal thinning are mandatory findings to diagnose keratoconus’ [3]. Nevertheless, there is evidence that keratoconus is characterized by marked degradation of the corneal extracellular matrix involving inflammatory features such as increased levels of MMP-9, IL-6, and TNF-α, as well as increased oxidative stress [4, 5]. Moreover patients with keratoconus have increased levels of inflammatory mediators in their tears, as shown in numerous studies [4, 5]. The role of inflammation induced by eye rubbing, which is a proven risk factor for keratoconus development, contact lens wear and ultraviolet irradiation, is another aspect of the inflammatory nature of keratoconus [4].

Whether the mere presence of those inflammatory markers in keratoconic corneas is a sufficient proof of inflammation, remains controversial. Could these biochemical findings possibly represent epiphenomena? And finally, is it justifiable to classify keratoconus as quasi-inflammatory or inflammatory- related condition? Future studies with large numbers of healthy eyes used as controls, and comparison of the levels of these markers in eyes with other inflammatory conditions in the cornea, will contribute to elucidate these questions.

In regard with the genetic components in keratoconus, although there is strong evidence for a genetic aetiology, the remarkable heterogeneity of the disease is impeding our understanding of its complex genetic nature [6, 7]. Genetic studies have focused on whole genome linkage and candidate gene analyses of keratoconus pedigrees. Scientists have discovered gene loci which may be involved in keratoconus. Specific candidate genes related to these loci have been repeatedly investigated, including the visual system homeobox gene 1 (VISX1), superoxide dismutase 1 (SOD1), lysyl oxidase (LOX), and multiple collagen genes (COL4A1-4) (COL5A1) [6, 7]. Thus far, results have been controversial, with some pedigrees demonstrating associations with mutations in these genes and others which did not reveal any association, again highlighting the genetic heterogeneity of the disease.

Other candidate genes including ZEB1, TGFB1, FLG, interleukin, and collagen may also play a role in the pathogenesis of keratoconus and require further investigation. However, it is unclear whether and how these gene mutations contribute to the pathogenesis of keratoconus. Similarly, genome-wide association studies and gene expression studies have been used in order to delineate the genetic mechanisms of keratoconus, but only with limited success. Hopefully next-generation sequencing technologies will encourage rapid progress in this field.

At the clinical level, corneal imaging technology evolved rapidly and generated tools, which enable the assessment of corneal biomechanics *in vivo.* Numerous studies showed that keratoconus is characterized by reduced corneal stiffening, resulting in a ‘weaker’ cornea [8, 9]. Corneal biomechanics have been introduced in the diagnostics of keratoconus with the hope that they will enable the detection of subclinical or *forme fruste* keratoconus. The major question is whether changes in the corneal geometrical features induce changes in corneal biomechanical properties or the opposite. Do biomechanical changes precede or follow the modification in corneal curvature, elevation and pachymetry? It has been suggested that corneal geometrical alterations are the secondary signs of keratoconus and that the earliest initial changes occur at the biomechanical level [10]. Theoretical analysis with finite element models supports the hypothesis that the initial biomechanical modification is focal in nature, rather than a uniform global weakening, and that the focal reduction in elastic modulus precipitates a cycle of biomechanical decompensation [10]. However, until the development of a screening system, which will detect and measure non-uniformity in biomechanical properties across the cornea and localize focal corneal weakening, this fundamental question will remain.

It was not until 1854 that British physician John Nottingham clearly described keratoconus and distinguished it from other ectasias of the cornea. Nobody could imagine that this corneal disorder would stand for a fascinating corneal mystery attracting the attention of scientists, clinicians and surgeons for more than 150 years. Basic and clinical research has enormously increased our understanding of this complex entity, but still important underlying mechanisms are yet to be investigated. Future developments in this field will definitely contribute to unravel the remaining mystery of keratoconus.

The current special issue on keratoconus is focused on recent advances in pathophysiology, diagnosis and management of the disease. Dr. Soiberman from Prof. Chakravarti's group at Wilmer Eye Institute presents a comprehensive update on the pathophysiology of the disease highlighting the latest research findings; while Dr. Moussa from Prof. Reitsamer's group is summarizing the current state of keratoconus genetics with special focus on the most recent discoveries. Prof. Ambrosio provides a valuable contribution to the literature sharing his innovative research findings in the field of corneal biomechanics, particularly in regards to keratoconus, and describing the implications of corneal biomechanics in refractive surgery. Professor Moschos describes the current practice in contact lenses for the management of keratoconus and Dr. Vastardis from Prof. Pajic's group summarizes the amazing advances in corneal crosslinking technology. The paper of Dr. Panos from Prof. Hafezi's group focuses on the corneal crosslinking for paediatric patients with keratoconus. The role of intracorneal ring segments in the management of keratoconus is thoroughly presented by Prof. Alio, who provided detailed, in-depth update on the topic. Subsequently Dr. Aiello from Prof. Maurino's group highlights the pearls and pitfalls in cataract surgery for patients with keratoconus in a very interesting article. Finally, Professor Seitz emphasizes on advances in penetrating keratoplasty for keratoconus, with special focus on excimer laser-assisted and femtosecond laser-assistant penetrating keratoplasty.
